# Static and Dynamic Compliance of Thick-Walled Vessel Models: Mathematical Modeling and Experimental Validation Using Silicone and Polyvinyl Alcohol (PVA) Hydrogel

**DOI:** 10.1007/s10439-026-04130-9

**Published:** 2026-04-07

**Authors:** Ashkan Shiravand, Elisabeth Janzen, Fabian Baumer, Wasilios Hatziklitiu, Giorgio Cattaneo

**Affiliations:** Institute of Biomedical Engineering, University of Stuttgart, Stuttgart, Germany

**Keywords:** Volumetric compliance, Thick-walled vessel model, Polyvinyl alcohol hydrogel (PVA-H), Viscoelasticity, Dynamic compliance, Aneurysm model

## Abstract

**Purpose:**

Thick-walled vascular models, including block models, are increasingly used for in vitro investigations of fluid dynamics and endovascular device testing, particularly fatigue testing at high frequencies, as valuable alternatives to thin-walled models due to their simpler fabrication. Physiologically compliant models provide more realistic insights into hemodynamics and implant deformation; however, no compact and experimentally validated closed-form equation exists to predict the volumetric compliance of thick-walled vessel models.

**Methods:**

A closed-form mathematical equation was derived to estimate the volumetric compliance of thick-walled cylindrical vessel models. The equation was experimentally validated through static compliance tests on cylindrical vascular models of varying wall thicknesses fabricated from two silicone elastomers and polyvinyl alcohol hydrogel (PVA-H) with four PVA concentrations (10, 12, 14, and 16 wt/vol.%). Dynamic compliance tests were performed at 1, 5, 10, and 30 Hz to assess the viscoelastic behavior of the materials. Finally, an applicability study was performed by fabricating block-form anatomical aneurysm models targeting physiological volumetric compliance based on the predicted equation.

**Results:**

Predicted and measured values showed strong agreement (*R*^2^ > 0.95). PVA-H-10 exhibited volumetric compliance within the physiological range (0.4–1.1%/mmHg). Volumetric compliance decreased by over 50% at 30 Hz, confirming viscoelastic behavior with a maximum loss to storage volumetric compliance ratio of 0.14. Measured aneurysm model compliance matched predictions with an error below 7%.

**Conclusion:**

This study provided a validated mathematical and experimental framework for fabricating thick-walled vascular models with physiologically relevant volumetric compliance and viscoelasticity for fluid dynamics and endovascular device testing.

## Introduction

Vascular compliance, defined as the change in vessel volume per unit change in transmural pressure, maintains continuous blood flow by smoothing the pulsatile nature of cardiac output [1]. It also governs pulse wave velocity [2] and influences hemodynamic loads exerted on the cardiovascular system and endovascular devices [3]. Vessel compliance has been shown to influence key fluid-dynamic quantities within the vessel, including velocity [4], flow patterns, and wall shear stress (WSS) [5]. It is thus essential for realistic in vitro studies of fluid dynamics, a crucial factor for initiation, progression, and treatment of vascular diseases such as atherosclerosis, stenosis, and aneurysms [3]. Furthermore, vessel compliance influences stent expansion, with low compliance restricting the full expansion of the stent, resulting in-stent underexpansion and an increased risk of in-stent restenosis [6].

Compliant and rigid vascular models have been widely used for investigation of fluid dynamics, method development, endovascular device testing, and physician training [7]. Polymeric models made from silicone elastomers [8, 9] and, more recently, from polyvinyl alcohol hydrogels (PVA-H) [10, 11] are particularly attractive due to their specific features, including tunable elasticity [12], friction [13], and optical transparency [14].

Mathematical and computational models are a well-established approach to relate applied loads to material deformation, therefore providing predictive insights that support experimental observations and validation of test setups [15–17]. In developing vascular models, a commonly used mathematical formulation is the Laplace law, which is used to predict the vessel wall stress and strain, and thus the diameter expansion as a result of internal pressure [18]. Under the thin-wall assumption, where wall thickness *s* is greatly smaller than vessel lumen diameter *D*_i_ (*s* ≪ *D*_i_), a uniformly distributed strain across the vessel wall can be assumed. This simplification results in a linear dependence of the vessel diameter change on the vessel diameter and the applied transmural pressure, and an inverse linear dependence on the wall thickness and elastic modulus of the vessel.

Despite advances in model materials and fabrication techniques, some crucial methodological challenges remain. First, when *s* ≥ 0.05*D*_i_, thin-wall assumptions are no longer reliable. Instead, the radial and circumferential stress distributions are properly described using Lamé’s equations which provide closed-form solutions for axisymmetric thick-walled cylinders under transmural pressure and form the basis for subsequent compliance derivations [19]. The latter is particularly relevant as most human arteries have thickness to diameter ratios greater than 5% [20]. However, the majority of studies used thin-wall formulations for estimation of compliance in cylindrical vessels even when wall thickness was not negligible relative to the lumen diameter [5, 15, 21–23]. Moreover, although physiological arterial walls are typically much thinner than the models investigated in this study, the present study focuses on thick-walled and block-type vascular phantoms, which are widely used in in vitro flow studies, endovascular device testing, and training applications due to their simpler and more robust fabrication by molding technique compared with anatomically thin-walled models with a defined wall thickness [24–26]. In such models, reproducing the bulk compliance response is often more relevant than replicating the exact anatomical wall thickness which introduces fabrication challenges.

Finally, since polymers such as silicones and hydrogels are viscoelastic, their compliance depends on frequency [27, 28]. This leads to a different pressure–volume curve of cylindrical models used in pulsatile fatigue testing of arterial implants at high frequencies. Since implant deformation must be comparable to that at physiological condition, based on ISO 25539-2:2020 and ASTM F2477:2023 international standards [29], there is the need to characterize and mathematically formulate the frequency-dependent compliance of vessel models, based on experimental data.

In this study, pursuing the goal of more reliable designing of in vitro cardiovascular studies and understanding of the results obtained, we address the following questions:

- Can the compliance of thick-walled models be reliably described using a closed-form mathematical equation?
- Is there a threshold in vessel wall thickness beyond which compliance does not change significantly, thereby providing a rational for the use of block models?
- Do common materials for in vitro testing undergo frequency-dependent compliance changes, and can this be assessed and accounted for fatigue testing purposes?

To answer these questions, we first derived a closed-form mathematical equation for estimation of the volumetric compliance of thick-walled cylindrical vessel models under static transmural pressure. The mathematical model was then validated by measuring the volumetric compliance of polymeric cylindrical vessel models fabricated from two distinct silicones and multiple PVA-H concentrations after the material’s elastic modulus had been characterized. Specimens with eight different wall thicknesses were investigated at both room temperature (RT) and physiological temperature 37 °C (PT). Moreover, the frequency-dependent behavior of vessel model’s compliance was quantified under sinusoidal dynamic loading at frequencies of 1, 5, 10, and 30 Hz to assess viscoelastic material properties. Finally, mathematical model was applied and in vitro compliance tests were performed using three block-form anatomical models. In summary, the key contributions of this work are a compact and validated formula for static volumetric compliance of thick-walled or block-type vessel models, a comparative dataset across silicones and PVA-H concentrations that can guide the selection and design of vascular phantoms with target compliance, and finally characterization of frequency-dependent compliance, considering the viscoelastic effects of model material, providing a guidance for future fatigue tests in cardiovascular field.

## Materials and Methods

### Derivation of Volumetric Compliance Formula for Thick-Walled Cylindrical Vessel Models

**Fig. 1:**
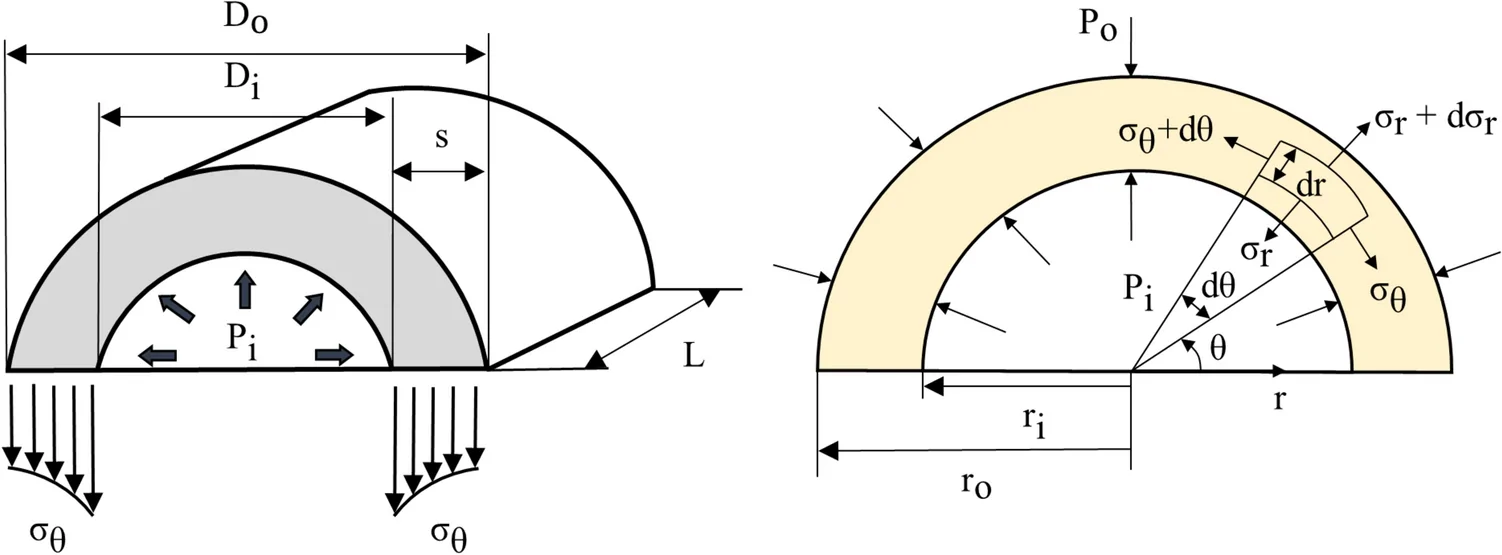
**a** Schematic of a thick-walled cylindrical vessel with inner diameter *D*_i_, outer diameter *D*_o_, and length *L*, subjected to an internal pressure *P*_i_ and an external pressure *P*_o_

A cylindrical thick-walled vessel with inner radius *r*_i_, outer radius *r*_o_, and wall thickness *s* is shown in Fig. 1. Since the vessel models were mounted between rigid tubes and were not closed by end caps in this study, the internal pressure did not generate classical axial pressure-thrust load on the vessel wall. Although the end fixations can cause constraint-induced axial stress, plane stress assumption was adopted in the axial direction and axial stress was neglected in the derivation of the equations (*σ*_*z*_ = 0) [30]. In contrast to the thin-walled cylindrical vessel assumption, in which the radial distension of the vessel results only from the circumferential strain (*ε*_*θ*_), both the radial strain (*ε*_r_) and *ε*_*θ*_ contribute to the overall radial distention in thick-walled cylinders. According to Lame’s theory of elasticity for thick-walled cylinders made of a homogeneous, isotropic, and linearly elastic material under static *P*_i_, the equations of radial (*σ*_r_) and circumferential stresses (*σ*_*θ*_) at a radial position *r* can be expressed as

1 $$\sigma_{{\mathrm{r}}} (r) = \frac{{P_{{\mathrm{i}}} r_{{\mathrm{i}}} }}{{r_{{\mathrm{o}}}^{2} - r_{{\mathrm{i}}}^{2} }}\left( {1 - \frac{{r_{{\mathrm{o}}}^{2} }}{{r^{2} }}} \right) - \frac{{P_{{\mathrm{o}}} r_{{\mathrm{o}}} }}{{r_{{\mathrm{o}}}^{2} - r_{{\mathrm{i}}}^{2} }}\left( {1 - \frac{{r_{{\mathrm{i}}}^{2} }}{{r^{2} }}} \right),$$

2 $$\sigma_{\theta } {(}r{) = }\frac{{P_{{\mathrm{i}}} r_{{\mathrm{i}}} }}{{r_{{\mathrm{o}}}^{{2}} { - }r_{{\mathrm{i}}}^{{2}} }}\left( {{1 + }\frac{{r_{{\mathrm{o}}}^{{2}} }}{{r^{{2}} }}} \right){ - }\frac{{{\mathrm{P}}_{{\mathrm{o}}} r_{{\mathrm{o}}} }}{{r_{{\mathrm{o}}}^{{2}} { - }r_{{\mathrm{i}}}^{{2}} }}\left( {{1 + }\frac{{r_{{\mathrm{i}}}^{{2}} }}{{r^{{2}} }}} \right).$$

In this study, *P*_o_ acting on the outer surface of the vessel is neglected assuming a traction-free outer surface, similar to previous studies [30]. Consequently, the vessel wall deformation results only from *P*_i_, which corresponds to the transmural pressure. Under these assumptions, the *σ*_r_ and *σ*_*θ*_ are given by

3 $$\sigma_{{\mathrm{r}}} (r) = \frac{{P_{{\mathrm{i}}} r_{{\mathrm{i}}} }}{{r_{{\mathrm{o}}}^{2} - r_{{\mathrm{i}}}^{2} }}\left( {1 - \frac{{r_{{\mathrm{o}}}^{2} }}{{r^{2} }}} \right) = \frac{{ - P_{{\mathrm{i}}} r_{{\mathrm{i}}}^{2} (r_{{\mathrm{o}}}^{2} - r^{2} )}}{{r^{2} (r_{{\mathrm{o}}}^{2} - r_{{\mathrm{i}}}^{2} )}},$$

4 $$\sigma_{\theta } (r) = \frac{{P_{{\mathrm{i}}} r_{{\mathrm{i}}} }}{{r_{{\mathrm{o}}}^{2} - r_{{\mathrm{i}}}^{2} }}\left( {1 + \frac{{r_{{\mathrm{o}}}^{2} }}{{r^{2} }}} \right) = \frac{{P_{{\mathrm{i}}} r_{{\mathrm{i}}}^{2} (r_{{\mathrm{o}}}^{2} + r^{2} )}}{{r^{2} (r_{{\mathrm{o}}}^{2} - r_{{\mathrm{i}}}^{2} )}}.$$

Tensile stresses and outward radial displacement were defined as positive throughout the derivation. Radial stress under internal pressure is therefore compressive. The circumferential strain *ε*_*θ*_, which results from the applied *P*_*i*_, can be computed as

5 $$\varepsilon_{\theta } = \frac{2\pi u(r)}{{2\pi r}} = \frac{u(r)}{r},$$

where *u*(*r*) is the radial displacement at radial position *r* due to circumferential deformation. Substituting Eq. (5) into Hook’s law relationship for a linearly elastic isotropic material, $\varepsilon_{\theta } { = }\frac{{1}}{E}\left( {\sigma_{\theta } - \nu \sigma_{{\mathrm{r}}} } \right)$, *u*(*r*) can be derived as

6 $$u{(}r{) = }\frac{r}{E}\left( {\sigma_{\theta } - \nu \sigma_{{\mathrm{r}}} } \right),$$

where *E* is the elastic modulus and *ν* is the Poisson’s ratio. Substituting Eqs. (3) and (4) into Eq. (5), *u*(*r*) can be written as

7 $$u{(}r{) = }\frac{{rP_{{\mathrm{i}}} r_{{\mathrm{i}}}^{{2}} }}{{E\left( {r_{{\mathrm{o}}}^{{2}} - r_{{\mathrm{i}}}^{{2}} } \right)}}\left[ {\left( {{1 } - \nu } \right){ + }\left( {{1 + }\nu } \right)\frac{{r_{{\mathrm{o}}}^{{2}} }}{{r^{{2}} }}} \right].$$

Substitution of the Lamé stress expressions into Hooke’s law yields a positive outward radial displacement, as expected for internal pressure. For perfectly incompressible materials with *ν* = 0.5, which is a reasonable assumption for elastomeric materials and hydrogels, the radial displacement at the inner wall (*r* = *r*_*i*_) can be written as follows:

8 $$u(r_{{\mathrm{i}}} ) = \frac{{P_{{\mathrm{i}}} }}{E}\left[ {\frac{{r_{{\mathrm{i}}}^{3} + 3r_{{\mathrm{i}}} r_{{\mathrm{o}}}^{2} }}{{2\left( {r_{{\mathrm{o}}}^{2} - r_{{\mathrm{i}}}^{2} } \right)}}} \right].$$

The inner volume of a cylindrical vessel with a length of *L* and inner radius of *r*_*i*_, as a function of *P*_*i*_, is given by

9 $$V(P_{{\mathrm{i}}} ) = \pi Lr_{{\mathrm{i}}} 2(P_{{\mathrm{i}}} ),$$

where the pressure-dependent inner radius is $r_{i} \left( {P_{i} } \right)\, = \,r_{i} \, + \,u\left( {r_{i} } \right)$. Substitution of Eq. (8) into Eq. (9) yields

10 $$V(P_{{\mathrm{i}}} ) = \pi L\left[ {r_{{\mathrm{i}}} + \frac{{P_{{\mathrm{i}}} }}{E}\left[ {\frac{{r_{{\mathrm{i}}}^{3} + 3r_{{\mathrm{i}}} r_{{\mathrm{o}}}^{2} }}{{2\left( {r_{{\mathrm{o}}}^{2} - r_{{\mathrm{i}}}^{2} } \right)}}} \right]} \right]^{2}.$$

Accordingly, volumetric compliance can be derived as a fractional change in volume per unit pressure from Eq. (10) as

11 $$C_{{\mathrm{v}}} = {\raise0.7ex\hbox{${\frac{{{\mathrm{d}}V}}{{{\mathrm{d}}P_{{\mathrm{i}}} }}}$} \!\mathord{\left/ {\vphantom {{\frac{{{\mathrm{d}}V}}{{{\mathrm{d}}P_{{\mathrm{i}}} }}} {V_{{\mathrm{o}}} }}}\right.\kern-0pt} \!\lower0.7ex\hbox{${V_{{\mathrm{o}}} }$}} = \frac{2}{E}\left( {\frac{{r_{{\mathrm{i}}}^{2} + 3r_{{\mathrm{o}}}^{2} }}{{2\left( {r_{{\mathrm{o}}}^{2} - r_{{\mathrm{i}}}^{2} } \right)}}} \right)\left[ {1 + \frac{{P_{i} }}{E}\left( {\frac{{r_{{\mathrm{i}}}^{2} + 3r_{{\mathrm{o}}}^{2} }}{{2\left( {r_{{\mathrm{o}}}^{2} - r_{{\mathrm{i}}}^{2} } \right)}}} \right)} \right],$$

where V_o_ is the initial (unloaded) vessel inner volume. In the experiments,* V*_o_ was calculated from the molded inner diameter and length of the vessel prior to testing. Under physiologically relevant conditions, the internal pressure is much smaller than the elastic modulus (*P*_i_ ≪ *E*). Consequently, the term $\frac{{P_{{\mathrm{i}}} }}{E}\left[ {\frac{{r_{{\mathrm{i}}}^{2} + 3r_{{\mathrm{o}}}^{2} }}{{2\left( {r_{{\mathrm{o}}}^{2} - r_{{\mathrm{i}}}^{2} } \right)}}} \right]$ ≪1, and *C*_v_ reduces to

12 $$C_{{\mathrm{v}}} = \frac{1}{E}\left[ {\frac{{r_{{\mathrm{i}}}^{2} + 3r_{{\mathrm{o}}}^{2} }}{{\left( {r_{{\mathrm{o}}}^{2} - r_{{\mathrm{i}}}^{2} } \right)}}} \right] \times 100 [\% /{\mathrm{mmHg}}].$$

Equation (12) provides a closed-form equation for the volumetric compliance of thick-walled cylindrical vessels under static loading as a function of vessel geometry and elastic modulus. For consistency with commonly reported physiological compliance values, the compliance is expressed as percentage change in volume per mmHg by multiplying by 100.

Equation (12) is based on the assumptions of linear elasticity and small deformation, indicating that the stress–strain relationship remains linear and that radial displacements are small relative to the vessel radius. Consequently, the proposed equation is valid where strains remain within the linear elastic regime, and it should not be extrapolated if the linear elastic regime is no longer valid.

From the analytical formulation, volumetric compliance depends on geometry through the dimensionless radius ratio *α* = *r*_o_/*r*_i_ (equivalently *α* = 1 + *s*/*r*_i_), rather than absolute wall thickness. To provide a practical and transferable design guideline for block-type vascular models, the onset of the steady-state regime was defined when the predicted volumetric compliance deviated by less than a prescribed tolerance *ϵ* = 10% from its asymptotic limit *C*_v,∞_ obtained for *α* → ∞,

13 $$\left| {\frac{{C_{{\mathrm{v}}} \left( \alpha \right) - C_{{{\mathrm{v}},\infty }} }}{{C_{{{\mathrm{v}},\infty }} }}} \right| < \in.$$

From Eq. (12), the volumetric compliance is given by

14 $$C_{{\mathrm{v}}} (\alpha ) \propto \left| {\frac{{1 + 3\alpha^{2} }}{{\alpha^{2} - 1}}} \right|$$

with an asymptotic limit of volumetric compliance $C_{{{\mathrm{v,}}\infty }} { = }\mathop {{\mathrm{lim}}}\limits_{\alpha \to \infty } C_{{\mathrm{v}}} {(}\alpha {) } \approx 3$. Substituting the asymptotic limit and Eq. (14) into Eq. (13) yields the following condition:

15 $$\alpha > \sqrt {1 + \frac{4}{3 \in }}.$$

Expressing this criterion in terms of wall thickness and inner radius gives

16 $$s > r_{{\mathrm{i}}} \left( {\sqrt {1 + \frac{4}{3 \in } - 1} } \right),$$

which provides a direct guideline for block-type vascular model design. For a tolerance of *∈* = 10%, this results in *s* ≈ 2.78*r*_i_, indicating that the wall thickness required to reach the steady-state compliance regime scales linearly with lumen radius.

### Model Preparation

Cylindrical vessel models with a length of 60 mm and an inner diameter of 4 mm were prepared, falling within the range of human carotid siphon diameters of 3 to 6 mm [31]. These models were fabricated with eight different outer diameters (6, 8, 14, 20, 25, 32, 40, and 50 mm) to provide a range of wall thicknesses for investigation of geometrical effect on volumetric compliance. Plexiglas^®^ tubes (Ernst Kienzle GmbH & Co. KG, Weinstadt, Germany) were used as casting molds, with 3D-printed rods of 4-mm diameter centrally positioned in each mold to produce hollow cylindrical vessel models. In addition to the casting molds used for preparation of the samples later employed in compliance measurements, five cylindrical test tubes (length = 100 mm, diameter = 7.6 mm) were used for preparation of tensile test specimens of each material. Two polymers were used in this study: silicone elastomers with two different formulations and PVA-H with different polyvinyl alcohol (PVA) concentrations.

A translucent silicone (Dragon Skin 10 NV, Smooth-On Inc., USA) and a transparent silicone (Solaris, Smooth-On Inc., USA), hereafter referred to silicone #1 and silicone #2, respectively, were used in this study. Each silicone consisted of two components (parts A and B), which were mixed in a 1:1 ratio using a spatula for 3 min, as recommended by the manufacturer. The mixtures were then subjected to vacuum degassing at −0.95 bar for 5 min to remove the entrapped air bubbles before casting. The degassed silicone #1 and silicone #2 were cast into the molds and cured at RT for 1.5 and 24 h, respectively, in accordance to manufacturer’s recommendation.

For the preparation of PVA-H models, a solvent mixture of dimethyl sulfoxide (DMSO) (Merck KGaA, Darmstadt, Germany) and distilled water at a ratio of 80:20 vol./vol.% was stirred at 300 rpm and heated to 80 °C using a hotplate stirrer (RCT basic, IKA-Werke GmbH & Co. KG, Germany), according to a protocol previously described [11]. Following the stirring of the solution for 5 min, PVA powder (Merck KGaA, Darmstadt, Germany) with a molecular weight of 85,000–124,000 g/mol and 99 + % hydrolyzation, was slowly added to the solvent mixture at concentrations of 10, 12, 14, and 16 wt/vol.%. The mixture was continuously stirred at 300 rpm and heated at 120 °C for 2–3 h until the PVA powder was completely dissolved and a transparent solution was obtained. The prepared solution was degassed in the vacuum chamber, as previously mentioned, for 10–15 min to eliminate air bubbles. The resulting hydrogel was cast into both molds described above and subjected to five freeze-thaw cycles, each consisting of freezing at −22 °C for 18 h and thawing at RT for 6 h to ensure a stable hydrogel network formation, according to our previous study [32].

### Compliance Measurement Setup

**Fig. 2:**
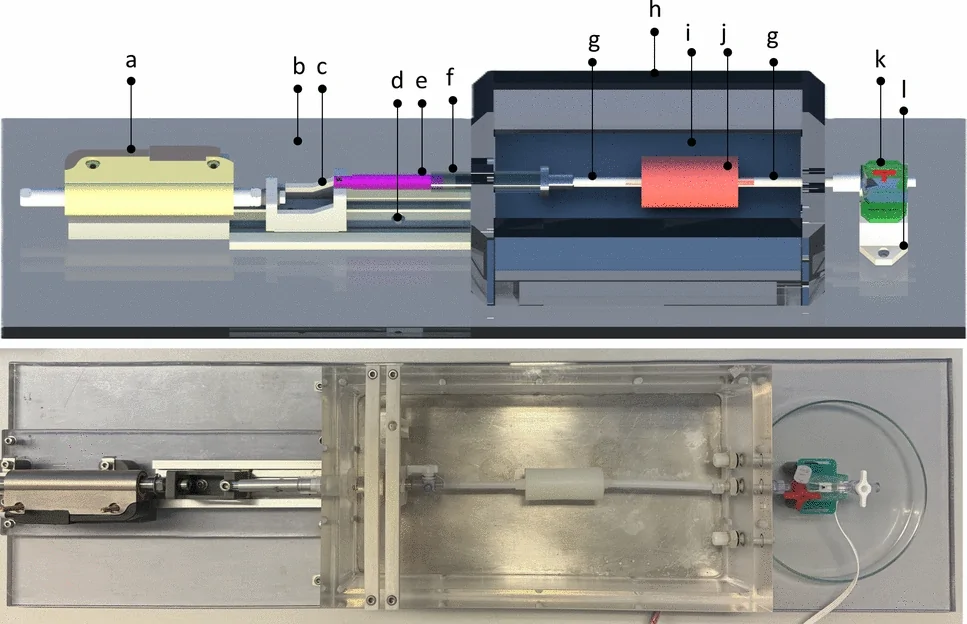
Compliance measurement test stand: **a** linear motor, **b** base plate, **c** connection and support component, **d** linear guide, **e** syringe plunge, **f** syringe barrel, **g** rigid tubes, **h** water basin, **i** aluminum heating plate, **j** vessel model, **k** pressure transducer, and **l** pressure transducer support

An in-house compliance measurement test stand (Fig. 2), previously described in detail by Shiravand et al. [32], was used to evaluate both static and dynamic compliance of the vessel models. In brief, a linear motor (NTI AG LinMot & MagSpring, Spreitenbach, Switzerland) was coupled to a 5-mL syringe (SETonic GmbH, Ilmenau, Germany) through a custom-designed 3D-printed connector, which enabled a precise and low-friction actuation of the syringe plunge along a linear guide (Igus GmbH, Cologne, Germany). The syringe was connected to the vessel model via a 3-way stopcock and rigid tubes.

A silicone heating foil (Thermo Flächenheizungs GmbH, Rohrbach, Germany) was attached to an aluminum plate and screwed to the bottom of the Plexiglas^®^ basin to heat the water basin and vessel model to PT. Pressure within the vessel models was continuously monitored by a disposable pressure transducer (Combitrans, B. Braun Melsungen AG, Melsungen, Germany) mounted on a 3D-printed holder at the distal end of the vessel model. The transducer signal was amplified into a measurable voltage between −10 and 10 V and acquired by a multifunction data acquisition device (National Instruments, Texas, USA). The whole set-up was mounted on a Plexiglas^®^ plate (800 mm × 300 mm) and controlled by LabVIEW software (National Instruments, Texas, USA). Both static and dynamic compliance tests were performed primarily at RT. Additional measurements at PT were performed to assess temperature sensitivity of the materials and are reported in the Supplementary Information (SI). For the latter, the test basin was filled with distilled water and its temperature was raised to 37 °C. A PID control system automatically regulated the voltage monitored by a temperature sensor (PT100, Promesstec GmbH, Schuettorf, Germany) to maintain the temperature of water basin at 37 °C ± 0.5 °C. The resulting hydrostatic pressure acting on the outer vessel surface due to the water column was on the order of a few millimeters of water with the maximum average value occurred for the thickest model with 23 mm thickness (< 30 mmH_2_O, corresponding to < 2.19 mmHg). This pressure was negligible compared to the applied internal pressure and was therefore neglected.

### Static Compliance Measurement

The pressure–volume relationship of the cylindrical vessel models was assessed using the compliance measurement test stand (Fig. 2). Five tests (*n* = 5) were performed for each specimen material (two silicones and four hydrogels) and eight thicknesses under both RT and PT, resulting in 480 tests in total, meaning that each specimen was tested five times. To minimize discrepancies between tensile and compliance measurements, all specimens were cast from the same batch of material and tested on the same day, reducing variability due to preparation differences and RT fluctuations. The basin was left empty at RT and filled with distilled water for measurements at PT. In the latter case, following the temperature stability of water basin, the specimens were placed in the basin for at least 1 h prior to testing to reach the thermal equilibrium. Static compliance (referred to 0 Hz) was determined by stepwise movement of the syringe plunge driven by the linear motor, leading to controlled volume increments in the vessel model.

First, the syringe was filled with distilled water from an external source (RT) or from the heated basin (PT) via a 3-way stopcock. Subsequently, the vessel models were connected to the rigid tubes and primed by multiple forward–backward piston movements to remove air bubbles and verify leakage-free connections. Static volumetric compliance was measured by advancing the motor in 0.05-mm steps, amounting to ~ 4 mm^3^ volume increments, with 0.5 s pause between the individual steps to ensure static condition. The pressure was recorded at each step simultaneously. The measurements were stopped when the maximum pressure of 130 mmHg was reached and a pressure–volume curve was obtained for each test. This procedure was repeated five times (*n* = 5) and the mean ± standard deviation (SD) was calculated.

Volumetric compliance (*C*_v_) was defined by normalizing compliance (*C*) to the diastolic volume (*V*_d_) as follows:

17 $$C_{{\mathrm{V}}} = \frac{C}{{V_{{\mathrm{d}}} }} = \frac{{V_{{\mathrm{s}}} - V_{{\mathrm{d}}} }}{{V_{{\mathrm{d}}} \times (P_{{\mathrm{s}}} - P_{{\mathrm{d}}} )}} \times 100 [\% /{\mathrm{mmHg}}],$$

where *V*_s_ is the systolic volume and *P*_s_ and *P*_d_ are the systolic and diastolic pressures, respectively. A pressure difference of 40 mmHg (80–120 mmHg), as recommended by [20] according to ANSI/ISO 7198:2016, was applied to calculate *C*_v_ based on Eq. (15). This protocol ensured reproducible measurements of static compliance under physiologically relevant loading conditions.

### Dynamic Compliance Measurement

Following each static compliance measurement, each specimen underwent dynamic compliance testing. For four PVA-H concentrations and two silicone materials, eight wall thicknesses were tested at both RT and PT. At each temperature, dynamic compliance testing was performed at four frequencies (1, 5, 10, and 30 Hz) sequentially. This sequential testing was repeated three times on the same specimen, yielding a total of 1152 dynamic tests. Frequencies of 1, 5, 10, and 30 Hz were selected to represent both physiological (1 Hz) [20] and extended dynamic conditions (5, 10, and 30 Hz), capturing viscoelastic dispersion of the material response. Due to the extensive number of measurements, dynamic tests were repeated three times per condition, compared to five times in static tests.

Motor displacement was calibrated to achieve the target intraluminal pressure at a frequency of 1 Hz. Accordingly, the diastolic and systolic pressures were set to 80 and 120 mmHg, respectively, as desired lower and upper limits for sinusoidal oscillation in the first step. To generate the sinusoidal calibration curve, the motor moved forward in 0.01 mm steps, amounting to 0.8 mm^3^ volume increments, with a pause duration of 100 ms between the individual steps, and the corresponding vessel pressure was recorded for each motor position. This process enabled the precise determination of the motor displacement and associated fluid volume corresponding to the target diastolic and systolic pressures at 1 Hz. In the second step, a sinusoidal input curve based on the calibration for each target frequency and test duration was generated. Following the generation of the signal, the motor oscillated at 1, 5, 10, and 30 Hz in sequence with the same volume displacement adjusted at 1 Hz. Thus, while systolic and diastolic pressure were defined at 1 Hz, deviations were allowed at other frequencies, depending on the viscoelastic material properties. To avoid artefacts from acceleration and deceleration of the motor, only five steady-state cycles (*n* = 5), excluding the first and last cycles, were selected for further analysis.

Since it was not possible to query the current position of the motor with sufficient temporal resolution, especially at higher frequencies, a linear potentiometer (LRW1-F, WayCon Positionsmesstechnik GmbH, Brühl, Germany) was integrated into the set-up to record the position of the motor for calculation of the total injected volume into the vessel model with high temporal resolution.

### Viscoelasticity

Under dynamic loading, the viscoelastic nature of the used hydrogel and silicone is supposed to lead to a frequency-dependent relationship between applied pressure and resulting volume change. In this study, the injected volume amplitude (∆*V*) was only calibrated at 1 Hz for each specimen and kept constant at higher frequencies. As a result, any change in the measured pressure amplitude Δ*P*(*ω*) directly reflects a frequency-dependent change in the effective stiffness and thus volumetric compliance of the vessel model. Accordingly, the magnitude of dynamic volumetric compliance *C*(*ω*) was defined as follows:

18 $$C(\omega ) = \frac{\Delta V}{{V_{{\mathrm{d}}} \times \Delta P(\omega )}} \, [{\mathrm{mL}}/{\mathrm{mmHg}}],$$

where ∆*P*(*ω*) is the pressure amplitude at a given frequency. The magnitude of dynamic compliance reflects the frequency-dependent stiffness of the vessel models.

Both pressure and displacement signals were acquired synchronously by the DAQ device with 2000 Hz sampling rate. The pressure transducer had a cut-off frequency of 3000 Hz, while the potentiometer had a cut-off frequency of 300 Hz. As both values are at least an order of magnitude higher than the maximum tested frequency (30 Hz), signal distortion and phase error due to instrumentation bandwidth were avoided. Therefore, phase lag *ϕ*(*ω*) between the volume and pressure signals was evaluated using fast Fourier transform (FFT) method. According to linear viscoelasticity theory [33], the frequency-dependent response can be expressed using a complex volumetric compliance C*(*ω*) as

19 $$C*\left( \omega \right) = \frac{\Delta V}{{V_{{\mathrm{d}}} \times \Delta P(\omega )}}e^{ - i\phi (\omega )} = C{ prime }(\omega ) - iC\prime \prime (\omega ),$$

where *C*′(*ω*) is the storage volumetric compliance, associated with elastic energy storage, and *C*″(*ω*) is the loss volumetric compliance, associated with viscous energy dissipation. These components were calculated as

20 $$C\prime \left( \omega \right) = \frac{\Delta V}{{V_{{\mathrm{d}}} \times \Delta P(\omega )}}\cos \phi (\omega ),$$

21 $$C\prime \prime \left( \omega \right) = \frac{\Delta V}{{V_{{\mathrm{d}}} \times \Delta P(\omega )}}\sin \phi (\omega ).$$

The ratio of loss to storage volumetric compliance *C*″(*ω*)/*C′*(*ω*) was then used as a dimensionless measure of viscous to elastic contribution of the material, similar to the loss tangent commonly used in dynamic mechanical analysis.

### Application Study on Anatomical Aneurysm Models

Three anatomical sidewall aneurysm models were selected and prepared to assess the applicability of this study for prediction of the volumetric compliance under anatomically realistic conditions. In contrast to simple models with constant radius, the selected models had varying effective radii along the parent artery, providing a more complex test for the compliance estimation.

**Fig. 3:**
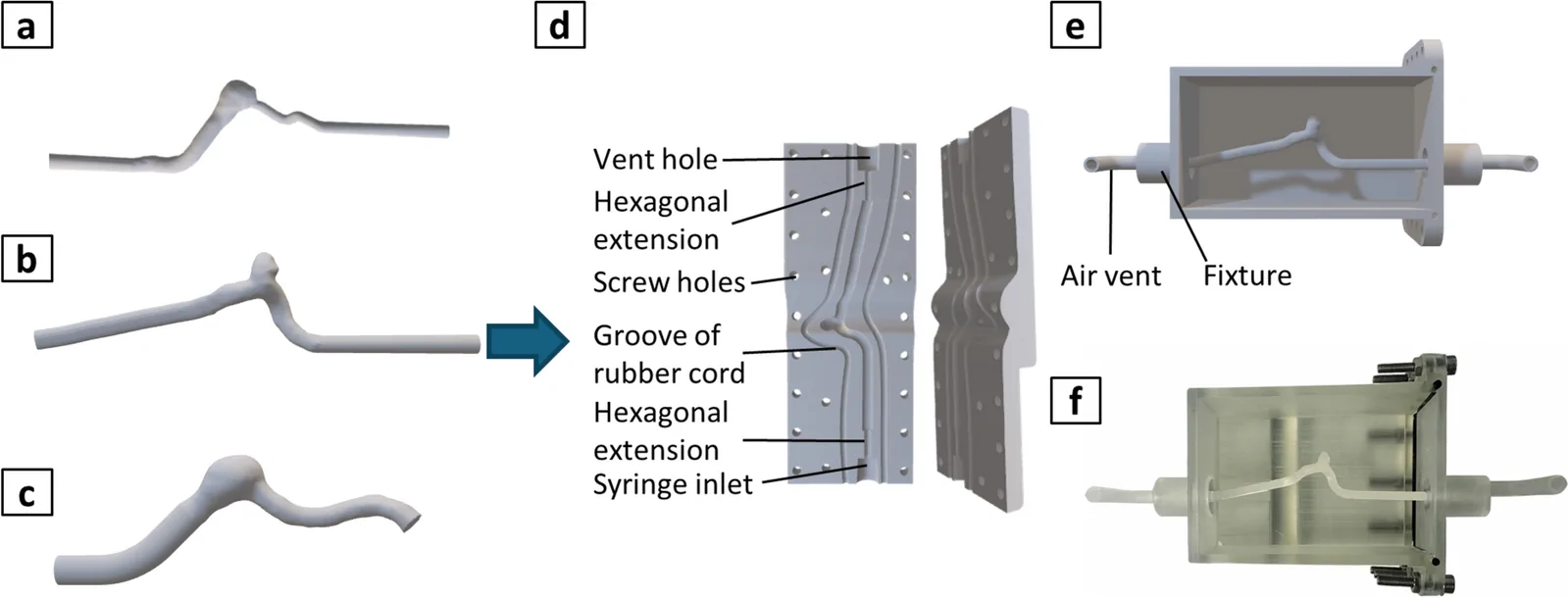
**a** 3D models of A1, **b** A2, and **c** A3. **d** 3D models of corresponding negative wax mold (**e**), PVA-H cast (**f**), as well as 3D-printed PVA-H cast with fixed wax lumen for A2

The first aneurysm model (A1) was taken from an anonymized patient previously studied by Ding et al. [25]. A1 is located at the vertebrobasilar artery with a neck diameter of 6.3 mm, and proximal and distal parent vessel diameters of approximately 1.69 mm and 3 mm, respectively (Fig. 3a). The second (A2) and third (A3) aneurysm models were obtained from the publicly available IntrA dataset repository [34]. A2 corresponds to a middle cerebral artery (MCA) aneurysm with a neck diameter of about 7 mm and a tortuous, S-shaped parent vessel geometry, with proximal and distal vessel diameters of approximately 3.6 mm and 3.2 mm, respectively (Fig. 3b). A3, positioned at the terminal segment of the ICA, characterized by a wider aneurysm neck of about 7.6 mm and proximal and distal vessel diameters of approximately 3.7 mm and 2.8 mm, respectively (Fig. 3c). Stereolithography (STL) files of the anatomical aneurysm models (A1–A3) were imported into Siemens NX (Version 2306, Siemens Digital Industries Software, USA) as solid body geometries for mold design.

Negative molds for fabrication of vessel lumens out of sacrificial wax were designed in Siemens NX, according to the methodology described in our previous study [32]. Briefly, each mold consisted of two halves with a syringe inlet for wax injection and vent holes for air release (Fig. 3d). Hexagonal extensions (5–10 mm in length) were added to both ends of the lumens for proper alignment and fixation of the vessel lumens within the subsequent casting molds. A 2-mm diameter rubber cord was placed into the designed grooves between the mold halves as a sealant. Additionally, screw holes were included to securely fasten the two mold halves with nuts and bolts.

Wax lumens were produced from a 1:1 (w/w) mixture of high-melting-point paraffin (HMPP) (327204, Sigma-Aldrich, Germany) and beeswax (BW) (5825.2, Carl Roth GmbH + Co. KG, Germany), following to our established protocol [32]. The molten wax mixture was injected into the negative molds and allowed to solidify at RT for 24 h.

Furthermore, cuboid molds (Fig. 3e and f) were designed and 3D printed using “Clear” resin for casting PVA-H around the wax lumens. The dimensions of the cuboid molds were 71.5 × 44 × 47 mm for A1, 82 × 43 × 37 mm for A2, and 50 × 38.3 × 32.4 mm for A3. These dimensions were selected to ensure a minimum distance between the lumen and the outer mold boundary exceeding 5 mm at all locations for achieving steady-state volumetric compliance. Fixtures with air vents matching the above-mentioned hexagonal extensions of the wax lumens were incorporated into the cuboid molds to ensure stable and proper positioning of the lumens during the casting process.

The average inner radii of the vessel lumens (*r*_i_), referred to effective radius, were measured using Vascular Modeling Toolkit (VMTK) module in 3D slicer software [35] and used for calculating the target elastic modulus from Eq. (12), aiming for a physiological volumetric compliance of approximately 0.5%/mmHg, consistent with the data of human cerebral arteries reported by Warnert et al. [36].

The selected concentration of PVA-H obtained from the calculation was prepared according to procedure described earlier and poured into three cuboid molds of each aneurysm (*n* = 3) and cylindrical test tubes (*n* = 5). The latter was used to verify the target elastic modulus. Wax lumens were subsequently dissolved by perfusing mineral oil at 55 °C through the lumen of the models. Each aneurysm model underwent five repeated pressure-volume measurements at RT, following the procedure described earlier, and the volumetric compliance was then calculated based on Eq. (15) and compared to the predicted value. Since the anatomical aneurysm geometries were derived from patient-specific CT images acquired at the diastolic phase and therefore represent a pressurized reference configuration rather than a fully unloaded state, the model size corresponds to pressurized state. To ensure consistency between the image-based geometry and the compliance calculation using the analytical formulation, volumetric compliance for the anatomical models was evaluated over a pressure increment of 40 mmHg (corresponding to the amplitude of physiological pulse pressure 80–120 mmHg) and normalized to diastolic reference volume. This approach avoids double-counting of pressure-induced deformation and effectively corresponds to a compliance defined with respect to a 0–40 mmHg pressure changes relative to the diastolic configuration, which corresponds to the resting state in the model.

### Statistics

The results of the experiments are presented as mean ± SD. Error bars on charts represent SDs. Microsoft Excel 16 (Microsoft Office 365, USA) was used to perform statistical data analysis using “Real Statistics Resource Pack” add-in. The graphs were plotted with QtiPlot (Version 0.9.9.13, Romania).

The Shapiro–Wilk test was performed to assess the normal distribution of the data. Two-way and one-way ANOVA tests were used to statistically determine the differences between different groups depending on the number of variables. Both tests were followed by *t* test for comparisons between individual groups. Statistical significance was defined as a two-tailed *p* value ≤ 0.05. Different letters (a, b, c, …) in charts indicate statistically significant differences between groups (*p* < 0.05). Outliers were included in the analysis, with their inclusion justified by their potential impact on the overall dataset.

Regression-based goodness-of-fit metrics were used to evaluate the agreement between the experimental data and the mathematical model. The coefficient of determination (*R*^2^) was calculated as

22 $$R^{2} = 1 - \frac{{\mathop \sum \nolimits_{i = 1}^{n} (u_{i} - \hat{u}_{i} )^{2} }}{{\mathop \sum \nolimits_{i = 1}^{n} (u_{i} - \overline{u})^{2} }},$$

where *u*_*i*_ is the experimental value, *û*_*i*_ is the model prediction, and *ū* is the average of the experimental values. The root mean square error (RMSE) was calculated as

23 $${\mathrm{RMSE}} = \sqrt {\frac{1}{n} \mathop \sum \limits_{i = 1}^{n} (u_{i} - \hat{u}_{i} )^{2} }.$$

And finally, the mean absolute error (MAE) was calculated as

24 $${\mathrm{MAE}} = \frac{1}{n} \mathop \sum \limits_{i = 1}^{n} \left| {u_{i} - \hat{u}_{i} } \right|.$$

Statistical significance of regression parameters was evaluated at a significance level of *p* < 0.05.

## Results

### Static Compliance

**Fig. 4:**
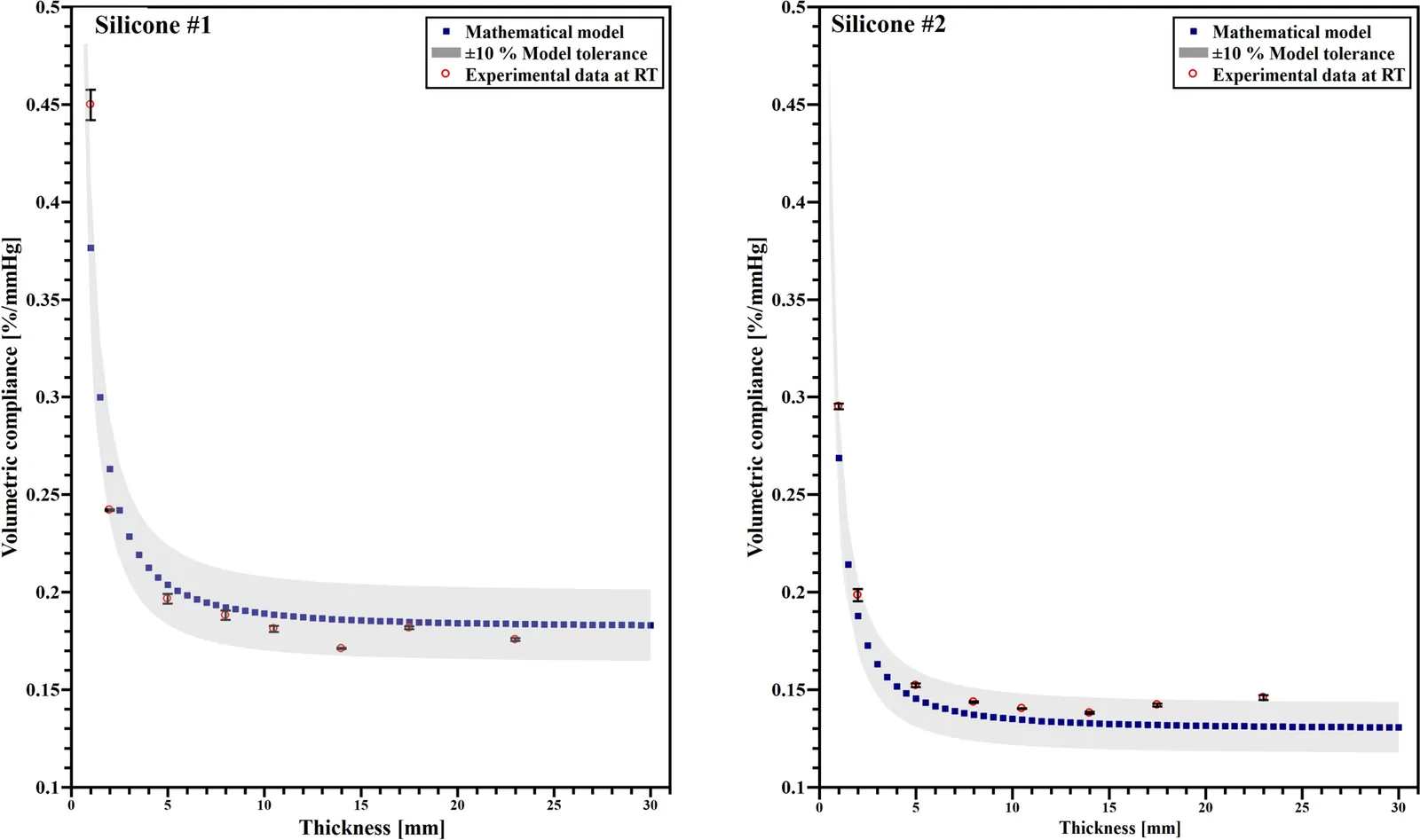
Volumetric compliance of silicone #1 and silicone #2 vessel models as a function of wall thickness at RT compared with the values calculated from the mathematical model (*n* = 5). Error bars represent SDs

Figure 4 demonstrates the measured volumetric compliance of both silicones across different wall thicknesses at RT as well as the values calculated according to the mathematical model (Eq. (12)) using the elastic modulus values provided in the SI. Table 1 summarizes the values of regression analysis results, including *R*^2^, RMSE, and MAE, to verify the agreement between experimental data and the mathematical model.

**Table 1:** Regression analysis results (*R*^2^, RMSE, MAE) comparing experimental and predicted volumetric compliance of silicone #1, silicone #2, and PVA-H samples at RT

Materials	*R*^2^	RMSE	MAE
Silicone #1	0.971	0.028	0.017
Silicone #2	0.995	0.012	0.010
PVA-H-10	0.955	0.077	0.062
PVA-H-12	0.968	0.070	0.042
PVA-H-14	0.966	0.023	0.018
PVA-H-16	0.994	0.036	0.019

As shown in Table 1, *R*^2^ values for both silicones indicate excellent agreement between measured and predicted values (*R*^2^ > 0.95). Both experimental and mathematical curves show a plateau in volumetric compliance for wall thicknesses greater than ~ 5 mm, amounting to ~ 0.19%/mmHg for silicone #1 and ~ 0.13%/mmHg for silicone #2. The lower volumetric compliance of silicone #2 compared to silicone #1 reflects its higher elastic modulus, as shown in SI. RMSE and MAE, as indicated in Table 1, each less than 10% of the mean experimental values, further confirming the strong agreement between experimental measurements and mathematical predictions.

The results for both silicones at PT are provided in the SI. Briefly, no statistically significant differences were observed between RT and PT elastic moduli (*p* > 0.05), and the experimental compliance values at PT remained in good agreement with the predictions of the mathematical model.

**Fig. 5:**
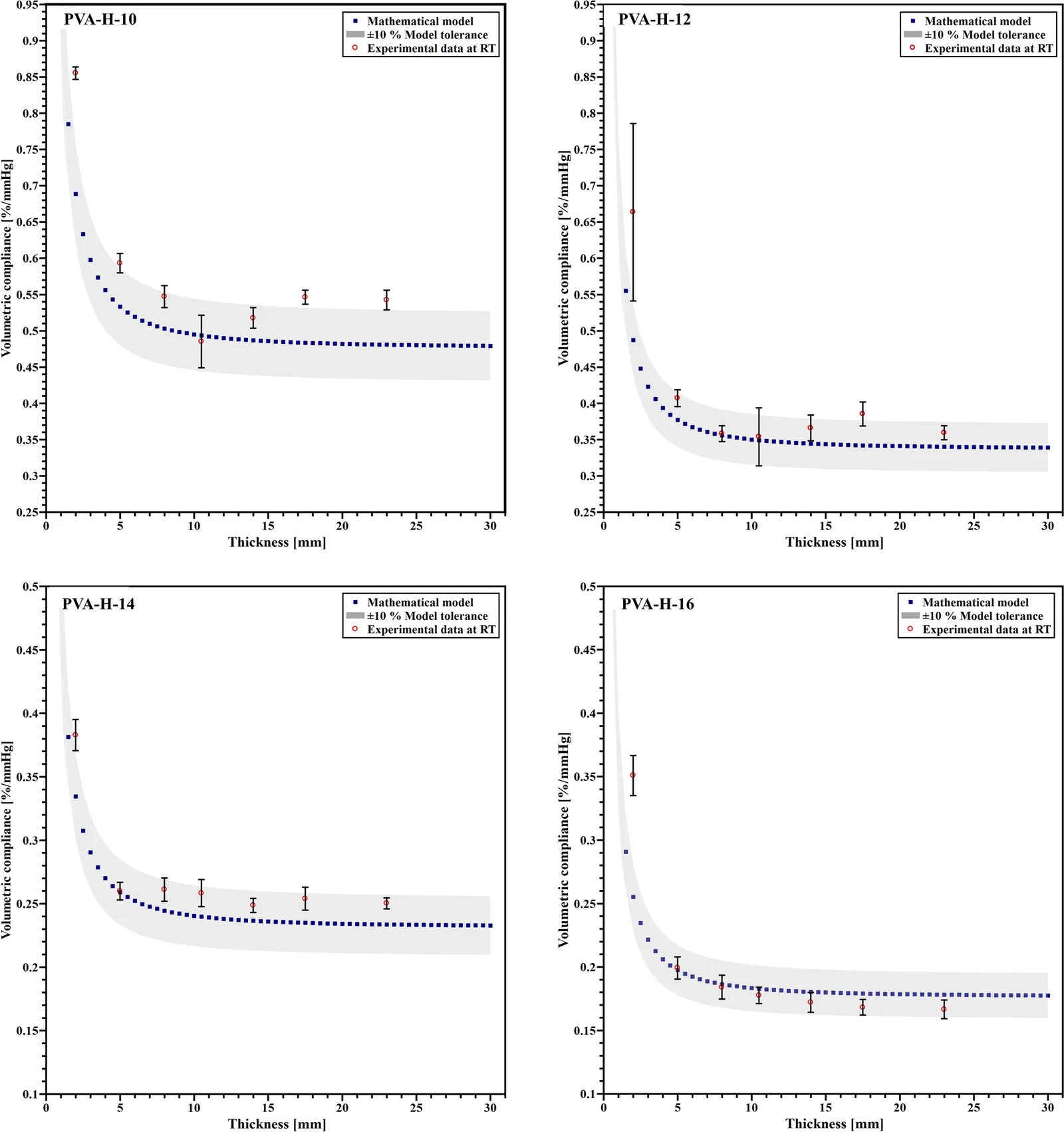
Volumetric compliance of PVA-H vessel models at different concentrations as a function of wall thickness at RT, compared with the values calculated from the mathematical model (*n* = 5). Error bars represent SDs

Figure 5 shows the measured volumetric compliance of PVA-H at different concentrations across different wall thicknesses at RT, as well as predicted values calculated from mathematical model. Since the target pressure difference of 80–120 mmHg could not be achieved for thickness of 1 mm due to material softness, the values of volumetric compliance could not be assessed. Both experimental and mathematical curves for all concentrations of PVA-H at RT show a decrease in volumetric compliance with increasing wall thickness, reaching a plateau in thicknesses above ~5 mm, with RMSE and MAE both less than 10% of the mean experimental values. Increasing PVA content led to a decrease in volumetric compliance values, from ~0.50%/mmHg for PVA-H-10 to ~0.18%/mmHg for PVA-H-16 at plateau, confirming the higher stiffness of higher concentration PVA-H, as shown in the results of tensile tests presented in SI. Similar to silicones, the mathematical model showed strong agreement with the experimental results, with *R*^2^ ranging from 0.955 to 0.994 within all concentrations. Nevertheless, compliance differences between experimental results and mathematical predictions were most pronounced at thickness of 2 mm, presumably due to PVA-H shrinkage.

The detailed volumetric compliance results of PVA-H samples at PT are provided in the SI. In summary, PT led to a statistically significant increase in compliance compared to room temperature (*p* < 0.05), while preserving the concentration-dependent trend of decreasing compliance with increasing PVA-H content.

The apparent transition to a steady-state regime, in which thickness-independent volumetric compliance was observed at wall thicknesses above approximately 5 mm (*α* = 3.78), is specific to the lumen radius used in this study. From the analytical formulation, volumetric compliance depends on geometry through the dimensionless ratio *α* = *r*_o_/*r*_*i*_ (or equivalently *s*/*r*_i_ = *α*−1), rather than absolute wall thickness.

The discrepancies between experimental data and model predictions were most pronounced at small thicknesses, presumably due to the shrinkage effects and temperature-induced microstructural changes in PVA-H network [32]. These discrepancies are primarily attributed to the increased sensitivity of volumetric compliance to geometric uncertainties when the wall thickness is low, where small absolute variations in inner or outer radius result in disproportionately large changes in compliance. Moreover, large deviations for thinner specimens may also reflect the nonlinear deformation, as these samples exceed the small-strain assumption inherent to the analytical model.

Despite localized deviations, particularly at lower wall thicknesses, the coefficient of determination (*R*^2^) remained high; however, *R*^2^ alone does not fully describe the agreement between experimental and predicted values. Therefore, additional regression-based goodness-of-fit metrics, including RMSE and MAE, were assessed to quantify the absolute prediction error. Both RMSE and MAE remained below 10% of the mean experimental values, indicating good overall predictive performance of the proposed model.

In summary, the results demonstrated a strong agreement (*R*^2^ > 0.95) between the experimental data and mathematical model for most of the tested samples, particularly for the thicknesses in plateau region. In all the materials, a reduction of compliance toward a plateau as a result of increase in wall thickness beyond ~5 mm was observed.

### Dynamic Compliance

**Fig. 6:**
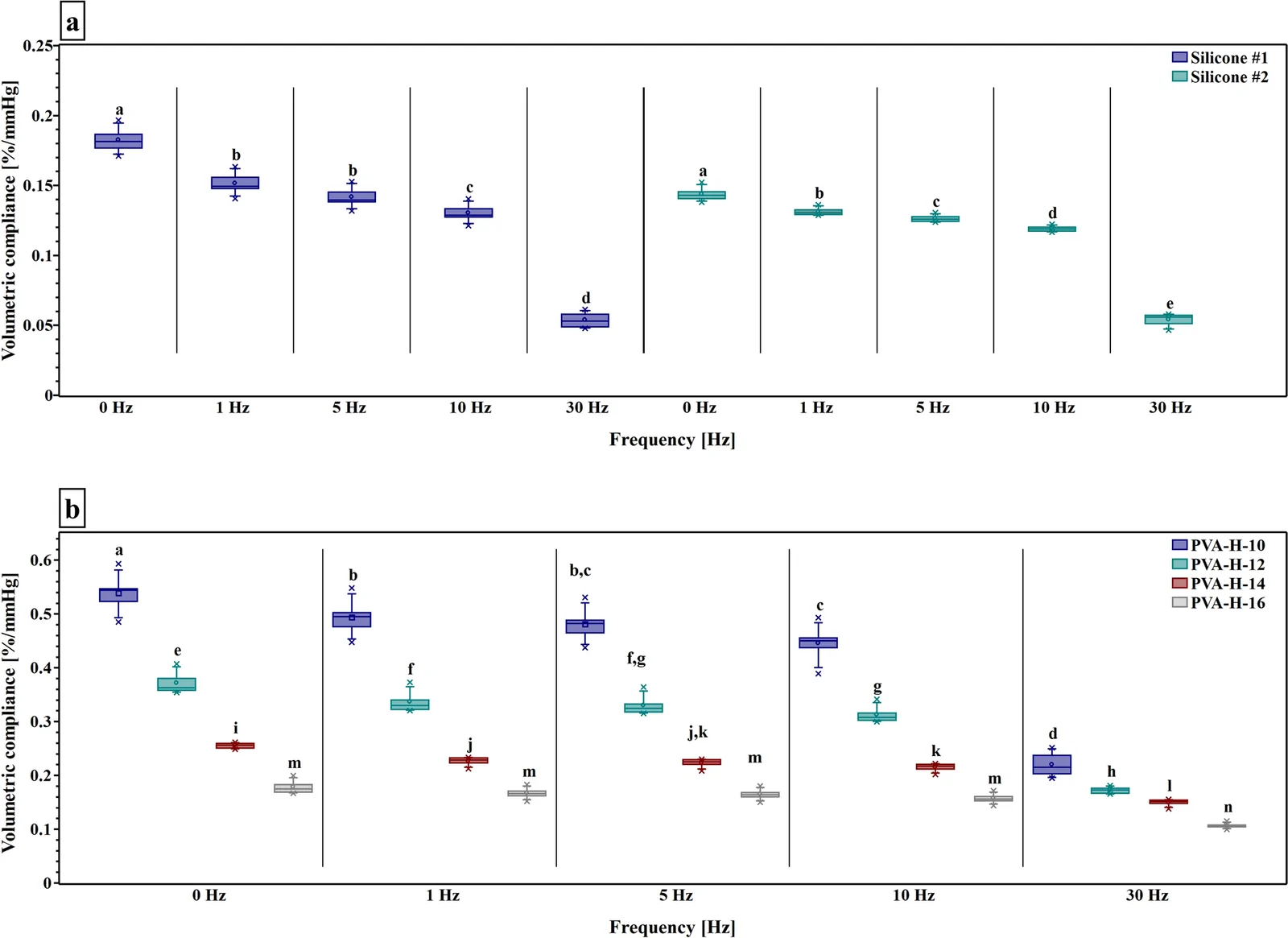
**a** Average volumetric compliance of silicone #1 and silicone #2 and **b** PVA-H at RT in the plateau region (*s*  ≥  5 mm) at different frequencies (0–30 Hz) (*n* = 6). Error bars represent SDs. Different letters (a, b, c, …) in charts indicate statistically significant differences between groups (*p* < 0.05)

The average volumetric compliance of silicone #1, silicone #2, and PVA-H at RT in the plateau region at frequencies of 0, 1, 5, 10, and 30 Hz is presented in Fig. 6. Single-factor ANOVA test indicated statistically significant differences (*p* < 0.05) between values of volumetric compliance at different frequencies for all materials, highlighting the frequency-dependent behavior of vessel models.

Volumetric compliance of both silicones decreased significantly with increasing frequency, reaching minimum values of ~0.05%/mmHg at 30 Hz (Fig. 6a). Overall, both silicones showed a consistent downward trend with frequency, with silicone #2 remaining less compliant than silicone #1 at all test conditions, confirming its higher elastic modulus.

Volumetric compliance of PVA-H at RT decreased with increasing frequency for all concentrations (Fig. 6b). Higher PVA concentrations exhibited less pronounced frequency-dependent changes, highlighting dominated elastic effects over viscous effects in stiffer PVA-H. For instance, PVA-H-16 showed no significant differences (*p* > 0.05) between different frequencies up to 10 Hz, with only 30 Hz indicating a significant decrease (*p* < 0.05), while PVA-H-10 showed a downward trend with significant differences at lower frequencies. The effect of frequency was particularly evident at 30 Hz especially for lower concentrations of PVA-H, where compliance values dropped by more than 50% compared to static (0 Hz) condition.

The detailed dynamic volumetric compliance results at PT are reported in the SI. In brief, all materials exhibited statistically significant frequency-dependent behavior (*p* < 0.05), while temperature had no significant effect on the dynamic compliance of silicones and led to a significant increase in the compliance of PVA-H due to thermal softening.

Overall, these results indicated the importance of considering viscoelastic effects when designing vascular models particularly for dynamic compliance testing and fatigue experiments of endovascular devices.

### Viscoelasticity

**Fig. 7:**
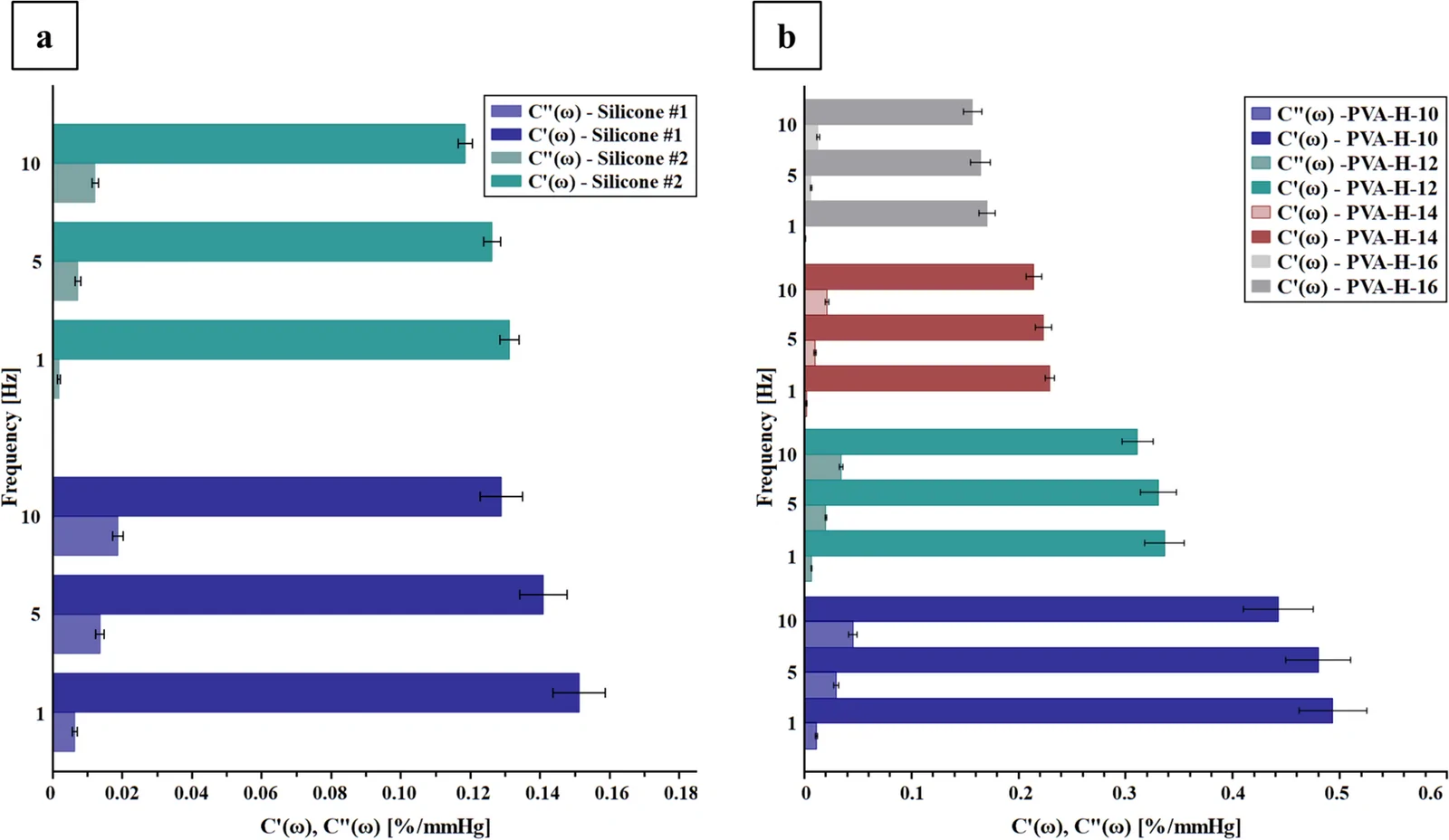
*C*′(*ω*) and *C*″(*ω*) for **a** silicone #1, silicone #2, and **b** different concentrations of PVA-H for different frequencies at RT (*n* = 3). Error bars represent SDs

Figure 7a demonstrates the average values of *C*′(*ω*) and *C*″(*ω*) for both silicones (*n* = 3). Phase lag values obtained using FFT analysis method were consistent and logically plausible up to 10 Hz. However, mixed and non-systematic phase lag values resulting from phase instability were obtained at 30 Hz, likely due to sampling limitations of the displacement signal (300 Hz) and possible inertial effects of the actuation system. Therefore, complex decomposition of volumetric compliance into *C*′(*ω*) and *C*″(*ω*) is presented for frequencies ≤ 10 Hz. This does not affect the assessment of the magnitude of dynamic volumetric compliance, which is computed from the ratio of volume and pressure amplitudes, since both sensors provided bandwidths well above the maximum excitation frequency.

One-factor ANOVA test for both silicones revealed statistically significant differences (*p* < 0.05) of both *C*′(*ω*) and *C*″(*ω*) across different frequencies, confirming the frequency-dependent viscoelastic response of the material. *C*′(*ω*) gradually decreased and *C*″(*ω*) increased slightly, showing a slight viscous contribution. The *C′*(*ω*)/*C*″(*ω*) ratio, presented in Table 2, ranged between 0.041 and 0.144 for silicone #1 and 0.013–0.102 for silicone #2, with the maximum value of ~0.144 observed for silicone #1 at 10 Hz, showing slightly higher damping effect.

**Table 2:** *C*″(*ω*)/*C*′(*ω*) ratios of different materials at different frequencies at RT

Materials	1 Hz	5 Hz	10 Hz
Silicone #1	0.041	0.096	0.144
Silicone #2	0.013	0.056	0.102
PVA-H-10 at RT	0.022	0.061	0.102
PVA-H-12 at RT	0.018	0.060	0.108
PVA-H-14 at RT	0.006	0.043	0.097
PVA-H-16 at RT	0.004	0.036	0.079

Similar frequency-dependent trend was observed for different concentrations of PVA-H at RT (Fig. 7b). Two-factor ANOVA test confirmed significant differences across both frequency and PVA concentration for *C*′(*ω*) and *C*″(*ω*). *C*′(*ω*) decreased and *C*″(*ω*) increased with increasing frequency, demonstrating increased viscous dissipation at higher frequencies. The effect was most pronounced for softer samples of PVA-H-10 and PVA-H-12, with the maximum *C*″(*ω*)/*C*′(*ω*) ratios of ~0.108 at 10 Hz. In contrast, PVA-H-14 and PVA-H-16 showed smaller ratios and thus a more pronounced elastic behavior.

Viscoelasticity results for PVA-H at PT, presented in the SI, show that while overall volumetric compliance increased at PT, the *C*″(*ω*)/*C*′(*ω*) ratio remained essentially unchanged, indicating a preserved viscoelastic balance.

Overall, all materials showed a viscous response, consistent with the results of dynamic compliance, highlighting the frequency-dependent behavior of the materials that must be accounted in vascular model design and fatigue tests of endovascular implants.

### Application Study on Anatomical Aneurysm Models

The measured average *r*_i_ of the three aneurysm models obtained using VMTK module in 3D slicer software were 1.26 ± 0.33 mm for A1, 1.53 ± 0.17 mm for A2, and 1.61 ± 0.40 mm for A3 (Table 3). Since the average *r*_i_ values for aneurysm models were nearly similar, a target elastic modulus of 0.08 MPa was calculated from Eq. (11) to achieve a physiological *C*_v_ of approximately 0.5%/mmHg in the steady-state region for all three aneurysm models. Therefore, PVA-H-10 with elastic modulus of 0.0839 ± 0.0071 MPa at RT was selected to prepare the aneurysm models and the corresponding cylindrical test tubes.

**Table 3:** Comparison of predicted and measured volumetric compliance (*C*_v_) of aneurysm models (A1–A3) with corresponding elastic modulus and percentage error

Aneurysm model	*r*_i_ [mm]	Measured elastic modulus [MPa]	Predicted *C*_v_ [%/mmHg]	Measured *C*_v_ [%/mmHg]	Percentage error [%]
A1	1.26 ± 0.33	0.0786 ± 0.0020	0.5137 ± 0.0061	0.5033 ± 0.0370	2.0232
A2	1.53 ± 0.17	0.0788 ± 0.0017	0.5130 ± 0.0062	0.4812 ± 0.0150	6.1976
A3	1.61 ± 0.40	0.0690 ± 0.0017	0.5886 ± 0.0104	0.5477 ± 0.0283	6.9599

The measured elastic moduli of the fabricated samples showed minor deviations from the reported elastic modulus in the SI, supposedly due to small variations in preparation process of PVA-H, particularly temperature fluctuations during thawing phase. As summarized in Table 3, the measured *C*_v_ values of A1 and A2 closely matched to the target physiological value of 0.5%/mmHg, while A3 showed a slightly higher compliance (0.5477%/mmHg) due to lower elastic modulus of 0.0690 ± 0.0017 MPa compared to the target value of 0.0839 ± 0.0071.

Overall, the agreement between the predicted and measured *C*_v_ with percentage error ≤ 7% demonstrated the applicability of the mathematical model for fabrication of physiologically compliant vascular models for cardiovascular research.

## Discussion

In this study, a closed-form mathematical equation for estimating the volumetric compliance of thick-walled cylindrical vessel models was derived and experimentally validated using silicone and PVA-H vascular models under static transmural pressure at RT and PT. The mathematical model reproduced the transition from a thickness-dependent to a steady-state region at approximately 5 mm wall thickness, as expected based on stress–strain relationship of thick-walled cylindrical vessels [30]. Specifically, normalized volumetric compliance depends on geometry through the radius ratio *α* = *r*_o_/*r*_i_ (or thickness ratio *s*/*r*_i_ = *α *− 1), rather than absolute wall thickness. This implies that the wall thickness required to reach thickness-independent compliance scales proportionally with lumen radius (*s* = 2.78*r*_i_), highlighting the applicability of the derived equation for fabrication of larger vessel models in block form. Regression-based goodness-of-fit metrics showed an excellent agreement between predicted and measured volumetric compliance within different materials, wall thicknesses, and temperatures, with *R*^2^ > 0.95 for nearly all the cases and RMSE and MAE metrics below 10%.

Silicone #2 showed lower volumetric compliance than Silicone #1, in line with its higher elastic modulus which further confirms the inverse proportionality between volumetric compliance and elastic modulus based on both the Laplace law [37] and the derived mathematical model in this study. Furthermore, the measured elastic modulus and volumetric compliance of both silicones were nearly identical at RT and PT, demonstrating the thermal stability of these materials within the tested temperature range, consistent with previous observations of Mahomed et al. for silicone elastomers [38]. In contrast, PVA-H samples showed a significant temperature dependency (*p* < 0.05), with the elastic modulus decreasing significantly at PT, resulting in higher volumetric compliance compared to RT. This result is consistent with previous findings on PVA-H, which reported that higher temperatures weaken hydrogen bonds between water molecules and polymer chains, leading to a reduction in tensile properties of PVA-H [10, 32, 39]. Furthermore, lower concentrations of PVA resulted in lower elastic modulus of PVA-H, confirming the mechanical tunability of PVA-H [12] for fabrication of thick-walled vascular models with physiological volumetric compliance. In the present study, the volumetric compliance of PVA-H-10 and PVA-H-12 at both RT and PT fell within the reported physiological range for human cerebral arteries (0.4–1.1%/mmHg) [36], showing the suitability of these formulations for realistic vascular modeling.

The results of dynamic compliance tests demonstrated a frequency dependence for all tested materials, with significant reduction of volumetric compliance as the frequency increased from 1 to 30 Hz, with reductions exceeding 50% at 30 Hz relative to static condition. This trend confirms the viscoelastic nature of the materials, where rapid loading limits molecular relaxation time and consequently increases apparent stiffness and reduces compliance [40]. This behavior is in agreement with previous studies which showed that the storage modulus of silicone rubbers and PVA-H increases with frequency using dynamic mechanical analysis [41–44]. Phase lag analysis further confirmed the frequency-dependent response of both materials, demonstrating a measurable delay between pressure and volume signals that increased with frequency, although the viscous damping showed small contribution in complex volumetric compliance with maximum *C″*(*ω*)/*C*′(*ω*) ratio of 0.14 occurred for silicone #1 at 10 Hz. These values are in line with those reported by Kosukegawa et al., who found loss to storage modulus ratios below 0.1 for PVA-H similar to the natural blood vessels [45], highlighting the physiological relevance of the tested models. Moreover, Kosukegawa et al. observed an increase in both storage and loss moduli with higher PVA concentrations resulting from enhanced degree of polymerization with minimal temperature dependence below 40 °C [45], consistent with the reduction of volumetric compliance as well as *C″*(*ω*)/*C*′(*ω*) in higher PVA concentrations at both RT and PT observed in the present study. This shows that higher concentrations of PVA results in stiffer material with lower viscous contribution.

Finally, an applicability study using anatomical aneurysm models showed an excellent agreement between predicted model and measured values with percentage error ≤ 7%, confirming the model potential in fabrication of thick-walled vascular models with physiological volumetric compliance and viscoelastic properties for realistic in vitro investigation of fluid dynamics and endovascular device testing.

Overall, a key finding of this study is that the volumetric compliance of the vessel models beyond a certain wall thickness depending on the lumen diameter (approximately 5 mm for the diameter used in our study) does not significantly change. This observation suggests that block vessel models can be mathematically described, enabling their use as simplified yet mechanically valid for applications where detailed wall geometry is not required. Since the wall thickness of even the aorta, the thickest human vessel, rarely exceeds 2 mm [46], the fabrication of accurate thin-walled and tortuous geometries is technically challenging, as it requires complex multi-part molds and precise demolding processes. In contrast, block-type or embedding models can be fabricated more easily using simple cuboid molds. The present findings confirmed that, for vessels with a diameter of 4 mm such as coronary and intracranial arteries [47], volumetric compliance enters a steady-state regime beyond approximately 5 mm wall thickness. In this regime, simplified block models can replicate the bulk mechanical response of vascular walls in terms of compliance by selecting an appropriate modulus and without needing anatomic wall thickness, therefore supporting realistic simulation of vascular behavior in fluid-dynamic and endovascular device testing applications. Additional major finding of this study is that the frequency-dependent reduction in volumetric compliance observed in dynamic tests suggests that implant fatigue testing at high frequencies must be calibrated for the applied pressure waveform to reproduce the correct volumetric or radial deformation corresponding to physiological conditions. Neglecting this effect could result in underestimation of device strain and lead to unrealistic fatigue performance predictions.

In this study, several limitations should be mentioned and addressed in the future. Although the developed model and experimental validation showed an agreement across materials and conditions, the derived equation assumes linear elasticity, isotropic material behavior, small deformation, and a constant Poisson’s ratio of 0.5 assuming an incompressible material. If these assumptions are reasonable for silicone and PVA-H within the small deformations range tested, both materials might exhibit slight compressibility and nonlinear stress–strain relationships at higher strains or during long-term cyclic loading, similar to natural blood vessels [37]. Furthermore, thinner and more compliant vessel models underwent larger strains, for which nonlinear elasticity and geometric nonlinearity need to be considered. Future work could extend the model by incorporating experimentally measured Poisson’s ratios and nonlinear elastic or hyperelastic constitutive terms to better capture the mechanical response of the materials, including natural blood vessels.

The analytical model assumes a traction-free outer boundary with external pressure *P*_o_ = 0. The outer surface of block-type vascular models may be partially constrained by surrounding material or housing in practice, which can restrict radial deformation and reduce the compliance relative to the free-boundary prediction. Nevertheless, as the wall thickness increases (*s* → ∞), both radial and circumferential stresses at the outer boundary asymptotically approach zero [19], indicating that the influence of external constraints diminishes in the thick-wall limit.

Furthermore, the vessel models were not closed by end caps and fixed at the ends without externally applied pre-stretch in this study. The inclusion of constraint-induced axial stress in the derivation of mathematical equation is expected to reduce predicted compliance; however, the excellent agreement observed in the thick-wall regime suggests that axial effects are second-order for the present configurations. Moreover, natural human arteries are subjected to a physiological axial pre-stretch which increases the circumferential stiffness and reduces compliance [48, 49]. As a result, the volumetric compliance values reported in this study may be overestimated compared to in vivo conditions and may therefore be interpreted as an upper bound for physiological compliance. Nevertheless, the absence of axial loading is assumed not to affect the validation of the mathematical equation as well as relative trends observed across different materials, wall thicknesses, temperatures, and loading frequencies, which remain valid for comparative and modeling purposes. A physiological axial pre-stretch should be applied and considered in future studies to further enhance physiological realism and enable direct assessment of its influence on static and dynamic compliance.

Another limitation of the present study is that the vessel models were fabricated from isotropic materials, while natural arteries exhibit anisotropic mechanical behavior resulting from their layered structure and oriented collagen fibers. This simplification neglects direction-dependent stiffness and fiber recruitment mechanisms that influence local strain distributions under pressure. However, the primary objective of this work was to enable derivation and experimental validation of a closed-form compliance model under simplified and well-controlled assumptions without introducing additional complexity associated with anisotropy. Future studies could incorporate anisotropic constitutive models or fiber-reinforced phantoms to better reproduce in vivo vessel mechanics.

A further limitation of this study was the inability to measure the compliance of the 1- and 2 mm PVA-H samples due to their softness which prevented achieving the target pressure range of 80–120 mmHg without causing sample excessive deformation which could not be predicted by analytical model due to the relatively small deformation assumption in the derivation of mathematical equation. This limitation does not affect the main conclusions because the focus was on thicknesses above 5 mm, where stable measurements were obtained. Additionally, the elastic modulus used in the mathematical model was calculated from the linear region of the experimental stress–strain curve rather than from the strains corresponding to 80–120 mmHg. Since the tested materials exhibited a nearly linear stress–strain response within this range, the deviation introduced by this assumption is expected to be minimal.

The inability to obtain reliable phase measurements at 30 Hz was likely due to signal limitations and sampling discrepancies between the pressure transducer (1300 Hz) and displacement potentiometer (300 Hz), rather than material behavior. Future setups should employ high-speed acquisition systems such as optical displacement sensors to overcome this limitation. Moreover, in addition to volumetric compliance, the assessment of local distensibility, displaying regional wall motion caused by pressure waves in anatomical vessels, should be studied using high-frame rate imaging systems. This system could be integrated into a mock circulatory loop with open end vessels to better simulate in vivo condition.

In this study, pressure was measured downstream of the compliant vessel segment, providing a global pressure signal representative of the average intraluminal pressure, rather than a local wall pressure at a specific axial location. Given the short length and relatively large diameter of the vessel, pressure gradients along the vessel were negligible, making downstream pressure measurement appropriate for volumetric compliance assessment. However, this configuration may introduce minor phase offsets at higher frequencies (30 Hz) due to fluid inertia and line dynamics between the vessel and the pressure sensor.

Environmental factors such as RT fluctuations (27 ± 2.3 °C), variations of RT during the thawing cycles of PVA-H, and PVA-H shrinkage particularly in thin models (1–2 mm) might have caused slight variability in the mechanical properties of the materials. Continuous monitoring and regulation of ambient temperature could minimize the RT fluctuations. Random measurements of PVA-H specimens confirmed measurable shrinkage in thin samples (≈ 10–15% thickness reduction), supporting shrinkage as a possible source of discrepancy at small wall thicknesses. Future studies should systematically evaluate the thickness deviations for all PVA-H samples using a statistically meaningful sample size to produce uncertainty range for compliance prediction, *C*_v_ (*s* ± Δ*s*). Furthermore, since water exposure has been shown to influence mechanical properties of PVA-H, differences between tensile tests performed in air-heating chamber and compliance tests performed in water-heating chamber at PT could introduce additional discrepancy. Future studies could incorporate an air-heating system to maintain PT during compliance measurements, therefore, reducing the effect of water absorption and providing a more consistent comparison between tensile and compliance tests. In this study, a closed-form equation for estimation of thick-walled cylindrical vessel models was successfully derived. The experimental validation of the equation was performed through extensive experimental static compliance tests on two silicones and four concentrations of PVA-H at both RT and PT. Among the tested materials, thick-walled vessel models made of PVA-H-10 and PVA-H-12 at RT and PT were found to be suitable for replication of physiological compliance. Dynamic compliance tests and phase lag analysis revealed the viscoelastic properties of both materials, replicating the time-delayed response of natural blood vessels. The applicability of this study was demonstrated by fabrication of thick-walled anatomical aneurysm models with realistic volumetric compliance. Overall, this study establishes a framework for the design and fabrication of thick-walled vascular models with physiologically relevant volumetric compliance and realistic viscoelastic behavior. As a future perspective, further research should explore and enhance the biomedical applications of such models, including their potential use in patient-specific fluid dynamic studies and advanced endovascular device testing under pulsatile flow conditions. The observed frequency-dependent compliance behavior highlights the importance of calibrating fatigue testing protocols for endovascular implants to reproduce physiological deformation.

## Data Availability

The data that support the findings of this study are openly available in “figshare” repository at https://dsgvo-gesetz.de/erwaegungsgruende/nr-26/.
